# Extracurricular activities and refugee students in Turkish educational settings: a systematic review

**DOI:** 10.3389/fpsyg.2026.1882378

**Published:** 2026-09-29

**Authors:** Esin Hazar

**Affiliations:** Faculty of Education, Sivas Cumhuriyet University, Sivas, Türkiye

**Keywords:** extracurricular activities, inclusion, refugee students, systematic review, Turkey

## Abstract

Turkey hosts the world’s largest refugee population, and extracurricular activities have increasingly been positioned as potential supports for refugee students’ psycho-social adjustment, school belonging, and peer integration. However, a comprehensive synthesis of ECA-related outcomes for refugee students in Turkish educational settings remains absent from the literature. This systematic review examined the associations between extracurricular activity participation and refugee student outcomes across psychological, social, and educational settings-related domains. Following PRISMA guidelines, studies were evaluated using the Mixed Methods Appraisal Tool (MMAT), and findings were integrated through the Systematic What Is Known (SWIM) framework. Findings suggest that ECAs are associated with potential improvements in school attachment, perceived social wellbeing, and aspects of psychological adjustment; however, outcomes varied substantially across studies and participant groups, and the overall certainty of evidence remained low to very low. The findings further suggest that many extracurricular activities operate primarily within an integration-oriented framework, producing individual-level benefits without consistently addressing relational and structural barriers to equitable participation. The review highlights the importance of culturally responsive facilitation, inclusion-oriented programme design, and greater attention to refugee students’ own perspectives in future research and practice. Overall, extracurricular activities appear promising but insufficiently understood mechanisms for supporting refugee students’ inclusion and psycho-social adjustment within educational settings.

**Systematic review registration:**

Open Science Framework (OSF), https://doi.org/10.17605/OSF.IO/EG4X8.

## Introduction

The Syrian civil war and the subsequent migration movements since 2011 have produced one of the most significant forced displacement crises in modern history, with Turkey hosting the world’s largest refugee population (International Labour Organization, 2023; United Nations High Commissioner for Refugees, 2023). For refugee children, this displacement is often accompanied by cumulative adversity, including trauma, poverty, and disrupted schooling (Carswell et al., 2011). While Turkey has made substantial policy commitments to education, structural constraints such as language barriers and inadequate teacher preparation for diversity continue to hinder effective participation (Çelik et al., 2023). Within this context, extracurricular activities (ECAs) have emerged as vital, yet under-theorized, mechanisms for supporting student wellbeing beyond the formal curriculum.

Extracurricular activities are defined in the broader literature as voluntary, non-credit events hosted by educational institutions outside regular instructional hours (Bartkus et al., 2012). An important qualification applies in the Turkish context reviewed here: national regulations state that students participate in at least one social or sports organisation (Millî Eğitim Bakanlığı, 2017), meaning participation is structurally required rather than freely self-selected. The psycho-social mechanisms of mandated versus genuinely voluntary participation may differ substantially; no included study assessed whether refugee students experienced ECA participation as voluntary or obligatory, and readers should bear this ambiguity in mind when interpreting reported outcomes. These activities, ranging from sports and music to school clubs, promote cognitive and emotional engagement by allowing students to develop physical and social skills in diverse social settings (Eccles et al., 2003; Lunenburg, 2010). For refugee and immigrant youth specifically, ECAs offer a unique space to cultivate a sense of belonging and civic participation (Serpil and Aksoy, 2026), develop interpersonal leadership and confidence, often serving as a bridge between their cultural identity and the host society (Mendenhall and Bartlett, 2018; Olliff, 2007).

The theoretical architecture of this review is underpinned by the conceptual tension between integration and inclusion (Vislie, 2003; Hausstätter and Jahnukainen, 2014). In the context of educational equity, this distinction is consequential: integration-oriented educational environments tend to place the burden of adaptation on the individual student, potentially leading to academic alienation and social isolation, whereas inclusion-oriented environments create structurally supportive conditions that foster sustainable engagement and institutional belonging. Inclusion calls for equity-oriented reforms that reconfigure the educational landscape to embrace diversity as an inherent pedagogical asset. Leveraging this dichotomy, the current synthesis critically interrogates whether ECAs in the Turkish context serve as instruments of structural integration—facilitating mere physical proximity—or as transformative inclusive practices that catalyze social justice, authentic belonging, and educational equity. While the empirical literature on refugee ECA participation predominantly relies on small-scale, non-randomized, and qualitative designs, applying the integration-versus-inclusion framework serves as an analytical heuristic rather than a rigid evaluative template. This lens enables us to critically interpret how micro-level empirical findings map onto macro-level systemic and structural outcomes. Furthermore, this theoretical lens accounts for developmental and institutional variations across educational levels—ranging from primary and secondary schools to tertiary settings—by examining how age-specific social needs, peer dynamics, and institutional structures shape refugee students’ experiences of inclusion.

In Turkey, while national regulations require student participation in at least one social or sports organization (Millî Eğitim Bakanlığı, 2017) as mentioned before, there is a notable absence of explicit equity provisions for refugee populations. Existing policies often delegate implementation to educational staff without providing targeted guidance on culturally responsive practices or linguistic supports (Sucu et al., 2024). This policy-practice gap suggests that while ECAs are structurally “integrated” into the educational system, their potential as “inclusive” tools remains empirically and theoretically underdeveloped in the Turkish literature.

Despite the growing interest in ECAs, a comprehensive synthesis of their effects on refugee students in Turkey is missing. This gap is particularly significant from an educational psychology perspective: the outcomes at stake—school belonging, socio-emotional adjustment, and peer integration—are fundamental to the psycho-social development of learners. This systematic review aims to fill this gap by addressing the following research questions:

RQ1. What are the perceived effects of ECAs on refugee students?

RQ2. What methodological characteristics define the existing body of research, and what gaps remain for future inquiry?

## Methods

This systematic review was conducted in accordance with the Preferred Reporting Items for Systematic Reviews and Meta-Analyses (PRISMA 2020) guidelines (Page et al., 2021). The review protocol was prospectively registered on the Open Science Framework (OSF) prior to data extraction and is publicly available at https://doi.org/10.17605/OSF.IO/EG4X8. The review evaluates outcomes not only in terms of whether ECAs are associated with measurable effects, but against the distributional question of for whom those effects occur and under what structural conditions. This evaluative stance is operationalized through the integration–inclusion framework (Vislie, 2003; Hausstätter and Jahnukainen, 2014), applied here as an interpretive lens rather than a definitive explanatory model; interpretive claims are consistently calibrated to the strength of the underlying evidence. To ground this framework methodologically rather than deploying it solely as a post-hoc interpretive lens, three framework-specific indicators were coded for each included study during data extraction and are reported in the ‘Framework Coding’ column of Table 1: (a) whether the ECA design was modified to facilitate refugee student participation (operationally defined as any explicit adaptation of content, language, or facilitation approach; coded N when adaptation was implied but not explicitly reported); (b) whether the facilitator received training in differentiated or culturally responsive pedagogy (operationally defined as explicit reporting of such training; coded N when absent or unreported, which was the case for all 15 studies); and (c) whether outcome data were disaggregated by participant background (refugee vs. host-country). Studies meeting none of these three criteria were coded I (integration logic); studies meeting at least one were coded I* (partial inclusion features present). To establish the reliability of this framework coding, the three indicators were applied independently by two reviewers—both experts in educational sciences—across all 15 included studies. Inter-rater agreement for the framework coding was calculated using Cohen’s kappa, yielding a *κ* = 0.81, indicating strong agreement (Cohen, 1988). Discrepancies occurred in two of 15 studies, both involving criterion (a), and were resolved through discussion and reference to the operational definitions above. As the results in Table 1 show, thirteen of fifteen studies were coded I and two (Güvenç, 2020; Sağın, 2022) were coded I* on the basis of criterion (c) alone, confirming that the evidence base overwhelmingly reflects an integration rather than inclusion paradigm.

**Table 1:** Data extraction of included studies.

Author/year	Design	Activity and duration	Sample (*n*)	Outcome measure(s)	Key result	MMAT/ES	Framework coding
Abay and Güllüpinar (2020)	Qualitative	Extracurricular sports & cultural activities (retrospective)	5 teachers, 15 principals (*n* = 20)	Barriers & strategies (inductive coding)	Social adaptation identified as main barrier; ECA recommended as strategy	Q: Y/Y/Y/Y/Y ES: N/A	I (None of (a)(b)(c))
Alkhlıf (2022)	Quant. Non-rand.	Badminton; 8 wks, 2×/wk	16 Turkish + 16 Syrian undergrads (*n* = 32)	Social Integration in Sports Scale (Yılmaz et al., 2006)	Social integration ↑ (*p* = 0.04); Turkish > Syrian post-hoc (*p* = 0.03)	QN: N/Y/Y/N/Y ES: not reported	I (None of (a)(b)(c))
Arısoy et al. (2022)	Quant. Non-rand.	STEM (Science, Technology, Engineering, and Mathematics)activities; 4 wks, 24 h total	10 Turkish + 10 Syrian primary (*n* = 20)	Walker-McConnell Social Competency Scale (Walker et al., 1991)	Social adaptation ↑ (*p* = 0.03)	QN: N/Y/Y/N/Y ES: not reported	I (None of (a)(b)(c))
Arslan and Görgülü (2025)	Qualitative	Various ECA (middle school teacher perspective)	10 middle school teachers (*n* = 10)	Semi-structured interview; content analysis	Teachers recommended ECA to address Syrian students’ adaptation	Q: Y/Y/Y/Y/Y ES: N/A	I (None of (a)(b)(c))
Bozdağ (2020)	Qualitative	Various ECA (retrospective, counselor perspective)	6 psychological counselors (*n* = 6)	Semi-structured focus group; thematic analysis	ECA cited as community-based protective factor for psychological resilience	Q: Y/Y/Y/Y/Y ES: N/A	I (None of (a)(b)(c))
Güvenç (2020)	Mixed Method	STEM activities; 10 weekly 2-h sessions	32 7th-grade Syrian refugees	School Attachment Scale (Hill, 2006); Attitude Toward School Scale (Alıcı, 2013)	School attachment ↑ (*p* = 0.04); Attitudes ↑ (*p* = 0.009); Teacher attachment ↑ (*p* = 0.005)	MM: Y/Y/Y/N/N ES: not reported	I* ((c) outcomes partially disaggregated)
Koşak and Atasoy (2022)	Qualitative	Poetry, painting, composition tournaments (retrospective)	18 school principals (*n* = 18)	Semi-structured interview; content analysis	Principals organised ECA to address Syrian students’ adaptation	Q: Y/Y/Y/Y/Y ES: N/A	I (None of (a)(b)(c))
Kök (2019)	Quant. Non-rand.	Basketball, archery, table tennis, dart; 8 wks, 4 h/wk	191 Syrian high schoolers (exp = 98, ctrl = 93)	Leisure Benefit Scale (Ho, 2008)	Physical (*p* < 0.001), psychological (*p* < 0.001), social wellbeing (*p* < 0.001) ↑ in exp. group	QN: N/Y/Y/N/Y ES: not reported	I (None of (a)(b)(c))
Özbek (2023)	Quant. Descriptive	Folk dance ECA (≥1 yr)	1,000 high school refugees	School Adaptation Scale; Social Integration in Sports Scale; Folk Dance Attitude Scale	No significant relationship between folk dance attitudes, school adaptation, or social integration in sports	QD: Y/N/Y/Y/Y ES: N/A	I (None of (a)(b)(c))
Sağın (2022)	Quant. Non-rand.	Mixed-nationality team sports; 10 wks	68 Turkish + 68 Syrian middle schoolers (exp = 68, ctrl = 68)	School Attachment Scale (Savi, 2011); Satisfaction with Life Scale (Gadermann et al., 2010)	School attachment ↑ (*p* = 0.001); Life satisfaction ↑ (*p* < 0.001) for Syrian exp. group	QN: N/Y/Y/N/Y ES: not reported	I* ((c) outcomes disaggregated by background)
Suna et al. (2021)	Qualitative	Various ECA (retrospective, counselor perspective)	19 school psychological counselors (*n* = 19)	Semi-structured interview; thematic analysis	Extracurricular social interactions key to reducing barriers and school adaptation	Q: Y/Y/Y/Y/Y ES: N/A	I (None of (a)(b)(c))
Tosun et al. (2018)	Quant. Descriptive	Various ECA (self-report participation)	83 high school refugees	Self-developed questionnaire on educational expectations & challenges	Most refugees had difficulty adapting; low ECA participation rate	QD: N/N/N/Y/Y ES: N/A	I (None of (a)(b)(c))
Yaşar et al. (2018)	Quant. Non-rand.	Group fitness, games, stretches; 10 wks, 2 h/wk	46 Turkish + 46 Syrian primary (exp = 46, ctrl = 46)	Matson Social Skills Assessment Scale for Children (MESSY; Matson, 1983)	Negative social behaviours ↓ for refugee students (*p* < 0.001); Prosocial behaviours NS (*p* = 0.96)	QN: N/Y/Y/N/Y ES: not reported	I (None of (a)(b)(c))
Yılgın et al. (2022)	Quant. Non-rand.	5 sports (table tennis, badminton, athletics, taekwondo, bocce-dart); 16 wks, coach-led	85 Turkish + 24 Syrian (9–15 yrs) (*n* = 109)	Social Adaptation Scale (Aydoğdu & Gürsoy, 2020)	Turkish students’ social adaptation ↑ > Syrian students’ (*p* = 0.028)	QN: N/Y/Y/N/Y ES: not reported	I (None of (a)(b)(c))
Yılmaz (2023)	Quant. Non-rand.	Dodgeball tournament; 8 weekly sessions	50 Syrian middle school refugees	Self-Concept Scale (Piers, 1984); Social Adaptation Scale (Aydoğdu & Gürsoy, 2020)	Self-esteem NS (*p* = 0.15); Social adaptation NS (*p* = 0.63)	QN: N/Y/Y/N/Y ES: not reported	I (None of (a)(b)(c))

ECA = extracurricular activity; Q = qualitative; QN = quantitative non-randomized; QD = quantitative descriptive; MM = mixed methods; Y = Yes; N = No; ES = effect size; N/A = not applicable; I = intervention/activity coding; I* = outcome disaggregated by group.

### Search strategy and information sources

A systematic literature search was executed through April 2026 across four electronic databases: Dergipark, Web of Science, Scopus, and the National Thesis Center of the Council of Higher Education (YÖK). The search covered the period from 2011 to 2026, to capture research emerging since the onset of the Syrian refugee crisis in Turkey. The English-language search string utilized was: (extracurricular OR cocurricular OR “non-classroom” OR “out of class” OR “after school” OR “social activity” OR “sports”) AND (effect OR impact OR outcome) AND (refugee OR “asylum seeker” OR “displaced”) AND (student OR pupil). Grey literature was actively sought to mitigate publication bias. This included systematic searching of the YÖK National Thesis Center for unpublished Master’s and doctoral dissertations—a particularly important source in the Turkish educational research context, where a substantial proportion of empirical work on refugee education is produced within graduate programmes and does not proceed to journal publication. The inclusion of grey literature followed best-practice guidance for systematic reviews (Lefebvre et al., 2022; McAuley et al., 2000; Gusenbauer and Haddaway, 2020), which consistently identifies unpublished and non-indexed sources as necessary to prevent the overestimation of intervention effects that results from publication bias toward statistically significant findings. It should further be noted that neither the English nor the Turkish search strings included activity-specific terms such as “school club,” “arts,” “music,” or “drama”; studies indexed under activity-specific rather than generic ECA terminology may have been missed, and this constitutes a boundary condition of the current synthesis that is acknowledged in the Limitations. A parallel Turkish-language search string was also applied to YÖK and Dergipark results: (ders dışı etkinlik OR okul kulübü OR spor OR sanat OR müzik) AND (etki OR sonuç) AND (mülteci OR sığınmacı) AND (öğrenci). Both strings were fixed prior to data collection and applied consistently. Search strings were tailored to the structural indexing characteristics of English and Turkish academic databases. In English databases, the term *“extracurricular”* functions as an established, comprehensive umbrella descriptor that effectively captures a wide range of non-academic activities (e.g., sports, arts, school clubs, and social programs). Conversely, in Turkish educational literature and database indexing, umbrella terms such as “ders dışı etkinlikler” are less consistently keywords-indexed. Therefore, to minimize the risk of omitting relevant non-indexed national literature, the Turkish search strings were explicitly expanded to include specific activity descriptors (e.g., *“okul kulüpleri,” “spor,” “sanat,” “müzik,” “sosyal faaliyetler”*).

### Inclusion and exclusion criteria

Studies were selected based on the following criteria:

1. Population: Refugee or asylum-seeking students from primary to tertiary education residing in Turkey.
2. Intervention: Structured and planned extracurricular activities (e.g., sports, arts, clubs) led by an instructor. Unstructured leisure activities were excluded to focus on intentional educational interventions.
3. Outcomes: Quantitative, qualitative, or mixed-method data reporting on physical or psycho-social wellbeing.
4. Publication Type: Peer-reviewed articles and dissertations/theses (Master’s and PhD).

Studies were excluded if they: (a) focused solely on non-refugee populations without a comparison group; (b) were not available in full text; or (c) consisted of non-empirical pieces such as editorials or opinion papers. No age restriction was applied to the population criterion, reflecting the conceptual scope of the review: extracurricular participation as a mechanism for social integration and wellbeing is relevant across the full age range of students in formal educational settings, including higher education. To provide a comprehensive synthesis of refugee students’ experiences, eligibility criteria were defined to encompass all formal educational settings in Turkey, ranging from primary and secondary schools (K-12) to tertiary/higher education institutions. Age restrictions were intentionally omitted from the population criteria to account for developmental transitions and the documented educational disruptions or age-grade mismatches frequently experienced by refugee populations.

### Study selection

Initial database searches yielded 211 potentially relevant citations across all databases. Following automated and manual deduplication, 202 unique records were identified for screening. The selection process was conducted in two distinct stages.

In Stage 1 (Title and Abstract Screening), two independent reviewers (the author and one academic with no financial or personal interest in any included study) assessed all records against the pre-defined inclusion and exclusion criteria. Records were classified as ‘include,’ ‘exclude,’ or ‘uncertain.’

Inter-rater agreement was calculated using Cohen’s kappa, yielding a κ of 0.84, which indicates strong agreement (Cohen, 1988). Two discrepancies were identified during Stage 1 and were resolved through structured discussion between the two reviewers, resulting in consensus.

In Stage 2 (Full-Text Review), the same two reviewers independently examined the full texts of all records retained from Stage 1. One discrepancy was identified during this stage and was resolved through structured discussion and re-examination of the predefined eligibility criteria, resulting in consensus. The final agreement at Stage 2 was *κ* = 0.83. No third adjudicator was required. A final set of 15 studies met all criteria for inclusion.

. This coefficient reflects agreement at the title and abstract screening stage; subsequent stages involved separate reviewer checks and consensus procedures. The PRISMA 2020 flow diagram illustrating the selection process is presented in Figure 1.

**Figure 1:**
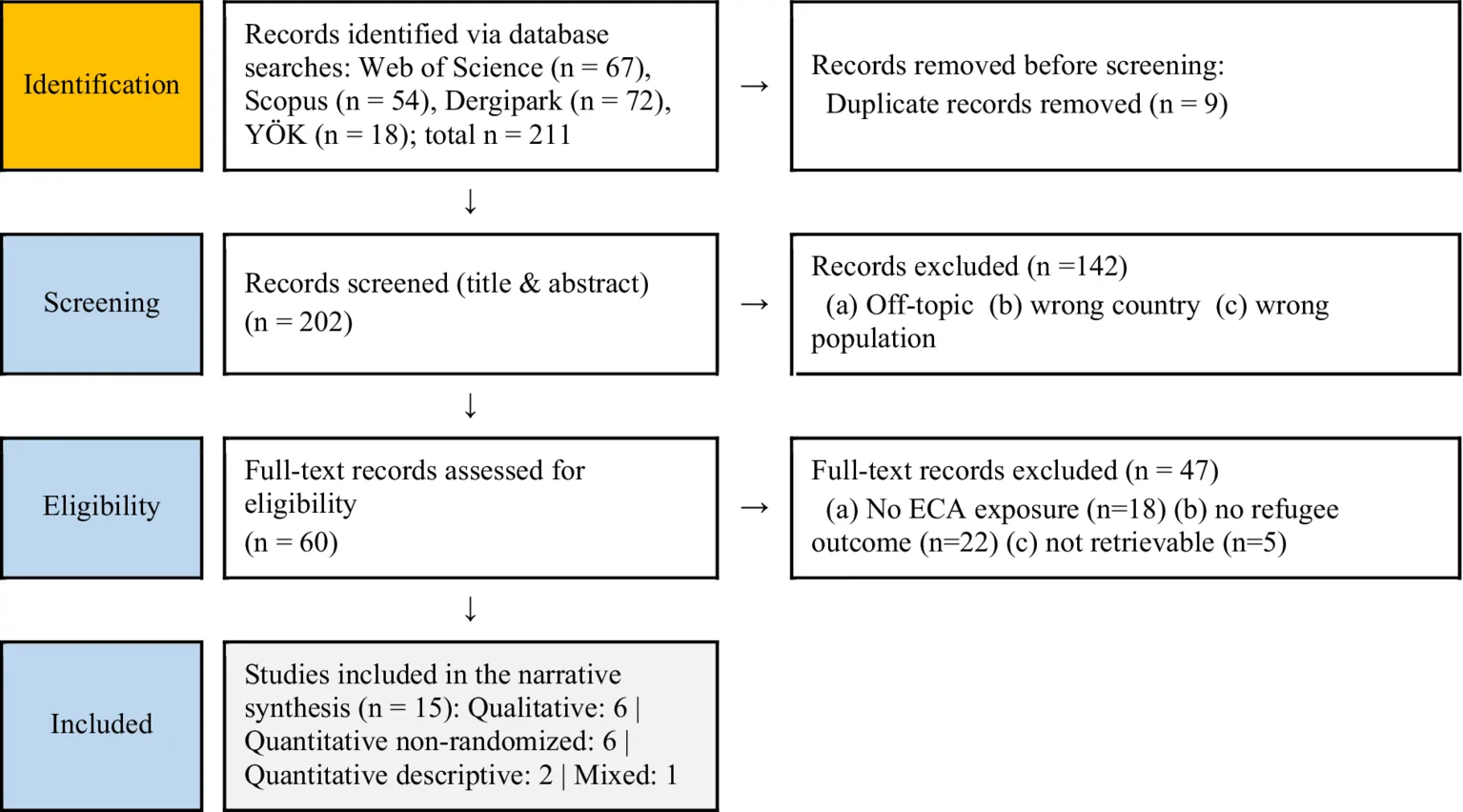
PRISMA 2020 flow diagram.

### Data extraction

Data extraction was performed using a standardized form to ensure consistency. In addition to the standard bibliographic and methodological fields listed below, the form included three framework-specific indicators aligned with the integration–inclusion distinction; the resulting codes are reported in the ‘Framework Coding’ column of Table 1. The following information was extracted from each study: (a) study design and methodological characteristics; (b) participant demographics (age, gender, ethnicity, and sample size); (c) type, duration, and frequency of the extracurricular activity; (d) outcome variables and measurement instruments; and (e) key quantitative results (means, standard deviations, *p*-values) and qualitative findings. Regarding quantitative data, effect sizes (Cohen’s d or η2) were recorded where available. To ensure the accuracy of the synthesis, a second reviewer independently verified the extracted data across the final study set, and any inconsistencies were resolved through collaborative review. Table 1 presents the data extraction summary for all 15 included studies.

### Quality assessment

The methodological quality of the included studies was independently appraised using the Mixed Methods Appraisal Tool (MMAT), Version 2018 (Hong et al., 2018). Each study was assessed by two independent reviewers to evaluate the appropriateness of the study design, data collection, and analysis techniques. Discrepancies were resolved through collaborative discussion. For quantitative studies, an additional risk of bias assessment was conducted across five domains: selection bias/group equivalence, validity of outcome measures, attrition, confounding variables, and reporting completeness. Each study was categorized as having Low, Moderate, or High risk of bias.

Following the recommendation of the MMAT developers, a total quality score was not calculated; instead, the detailed ratings per domain are presented in Table 2. All 15 studies were retained regardless of their quality rating to avoid further limiting the sparse evidence base in this emerging field. However, these quality ratings were used to contextualize the findings and provide a more cautious interpretation of the results. Güvenç (2020) appears twice in Table 2 because it employs a mixed-methods design: MMAT guidelines recommend applying the relevant single-design criteria to each component separately (Hong et al., 2018). Accordingly, the quantitative component was appraised against the non-randomized quantitative criteria (N1–N5) and the mixed-methods component against the mixed-methods criteria (M1–M5). This dual appraisal allows readers to evaluate the quality of each strand independently.

**Table 2:** Quality assessment of included studies.

Study	MMAT criterion	C1	C2	C3	C4	C5	Overall risk
Abay and Güllüpinar (2020)	Q1: Research Q clear | Q2: Data adequate | Q3: Findings from data | Q4: Interpretation correct | Q5: Coherence	Y	Y	Y	Y	Y	Low
Arslan and Görgülü (2025)	Q1: Research Q clear | Q2: Data adequate | Q3: Findings from data | Q4: Interpretation correct | Q5: Coherence	Y	Y	Y	Y	Y	Low
Bozdağ (2020)	Q1: Research Q clear | Q2: Data adequate | Q3: Findings from data | Q4: Interpretation correct | Q5: Coherence	Y	Y	Y	Y	Y	Low
Koşak and Atasoy (2022)	Q1: Research Q clear | Q2: Data adequate | Q3: Findings from data | Q4: Interpretation correct | Q5: Coherence	Y	Y	Y	Y	Y	Low
Suna et al. (2021)	Q1: Research Q clear | Q2: Data adequate | Q3: Findings from data | Q4: Interpretation correct | Q5: Coherence	Y	Y	Y	Y	Y	Low
Alkhlıf (2022)	N1: Comparable groups | N2: Intervention clear | N3: Outcome complete | N4: Confounders controlled | N5: Validated outcomes	N	Y	Y	N	Y	High
Arısoy et al. (2022)	N1: Comparable groups | N2: Intervention clear | N3: Outcome complete | N4: Confounders controlled | N5: Validated outcomes	N	Y	Y	N	Y	High
Güvenç (2020)—quant	N1: Comparable groups | N2: Intervention clear | N3: Outcome complete | N4: Confounders controlled | N5: Validated outcomes	N	Y	Y	N	Y	High
Kök (2019)	N1: Comparable groups | N2: Intervention clear | N3: Outcome complete | N4: Confounders controlled | N5: Validated outcomes	N	Y	Y	N	Y	High
Sağın (2022)	N1: Comparable groups | N2: Intervention clear | N3: Outcome complete | N4: Confounders controlled | N5: Validated outcomes	N	Y	Y	N	Y	High
Yaşar et al. (2018)	N1: Comparable groups | N2: Intervention clear | N3: Outcome complete | N4: Confounders controlled | N5: Validated outcomes	N	Y	Y	N	Y	High
Yılgın et al. (2022)	N1: Comparable groups | N2: Intervention clear | N3: Outcome complete | N4: Confounders controlled | N5: Validated outcomes	N	Y	Y	N	Y	High
Yılmaz (2023)	N1: Comparable groups | N2: Intervention clear | N3: Outcome complete | N4: Confounders controlled | N5: Validated outcomes	N	Y	Y	N	Y	High
Özbek (2023)	D1: Sample representative | D2: Response bias addressed | D3: Outcomes valid | D4: Analysis appropriate | D5: Conclusions match data	Y	N	Y	Y	Y	Moderate
Tosun et al. (2018)	D1: Sample representative | D2: Response bias addressed | D3: Outcomes valid | D4: Analysis appropriate | D5: Conclusions match data	N	N	N	Y	Y	High
Güvenç (2020)—mixed	M1: Rationale for MM | M2: Integration evident | M3: Divergence addressed | M4: Mixed-method inference | M5: Limitations acknowledged	Y	Y	Y	N	N	Moderate

Q = qualitative criteria; N = non-randomized quantitative criteria; D = descriptive quantitative criteria; M = mixed-methods criteria. Y = Yes; N = No. Güvenç (2020) is assessed twice per MMAT guidelines because it employs a mixed-methods design; both strands are appraised separately.

Table 2 presents the MMAT quality appraisal. All non-randomized quantitative studies failed MMAT criterion N1 (comparable groups/absence of selection bias), reflecting the absence of random allocation. Seven of eight also failed N4 (confounders controlled), as none reported analysis of potential confounders such as language proficiency, length of residency, or socioeconomic status. Risk-of-bias ratings are provided in the final column; eight of 15 studies were rated as High risk. These quality ratings were not treated as a binary filter but were integrated into the synthesis in three substantive ways. First, they informed the GRADE (Grading of Recommendations Assessment, Development and Evaluation)-adapted certainty ratings assigned to each outcome domain in the Findings section: no outcome domain was rated above Low certainty, and most were rated Very Low, primarily due to the pervasive high risk of bias and the near absence of effect size reporting. Second, they calibrated the degree of interpretive confidence applied to specific findings: positive outcomes from studies rated High risk are described as preliminary or directional rather than as established effects, and the language of conclusions was intentionally aligned with the evidentiary strength of the included studies. Third, the overall quality pattern itself became an analytic observation: the combination of widespread high risk of bias and limited statistical reporting suggests that much of the existing literature has approached ECAs primarily as beneficial interventions rather than as programmes requiring rigorous equity-oriented evaluation. This observation directly informed the review’s recommendations for future research design.

Because the included evidence base consisted predominantly of heterogeneous non-randomized and mixed-method studies, the review employed a GRADE-adapted, narrative-oriented certainty framework tailored to the characteristics of the available evidence intended to support interpretive transparency rather than formal evidence grading. The adaptation retained the core principle of explicitly judging confidence in the synthesized findings while accommodating the predominantly qualitative and heterogeneous nature of the evidence base. Certainty judgments considered methodological limitations/risk of bias, consistency of findings, indirectness, and the adequacy and relevance of the supporting evidence. Publication bias was also considered, particularly given the predominance of small, non-randomized studies and the limited reporting of null or adverse findings. Potential upgrading considerations were reviewed but were not applied because the available evidence did not provide sufficient grounds for increasing certainty. Associations observed in the included studies therefore cannot be interpreted causally and may reflect pre-existing group differences, maturation effects, selection effects, or unmeasured contextual factors.

### Data synthesis

A meta-analysis was not feasible due to the substantial heterogeneity in study designs (quantitative, qualitative, and mixed-methods), diverse outcome measures, and inconsistent reporting of effect sizes. Consequently, a narrative synthesis was conducted following the SWIM (Systematic What Is Known) framework (Gough et al., 2012), which organises evidence through three analytic steps: (a) mapping the landscape of available studies; (b) synthesising findings across heterogeneous designs; and (c) interpreting the meaning and applicability of the evidence in relation to the review question. These steps were operationalised as follows. The landscape mapping step is realised through Table 1 (data extraction by study) and Table 3 (narrative synthesis by outcome domain), which together provide a structured overview of study characteristics, design distribution, and the range of outcomes examined. The cross-design synthesis step is conducted outcome-domain by outcome-domain in the Findings section, grouping quantitative, qualitative, and mixed-methods evidence addressing the same construct and examining points of convergence or divergence across designs. The interpretive step is conducted in the Discussion section, where integrated findings are evaluated against the integration–inclusion normative framework to assess the practical and theoretical significance of the evidence in relation to the review’s research questions. The certainty of evidence was assessed using a simplified four-level scale (High, Moderate, Low, Very Low) adapted from the GRADE approach. This adaptation retained the four primary downgrading criteria (risk of bias, inconsistency, indirectness, imprecision) while omitting upgrading criteria (inapplicable given non-experimental designs) and treating publication bias separately in Limitations; this selective application represents a deviation from the full GRADE protocol and certainty ratings should be interpreted accordingly (Guyatt et al., 2008). Certainty ratings were determined by evaluating four downgrading criteria: (1) risk of bias (assessed via MMAT and the additional risk-of-bias appraisal described above); (2) inconsistency (degree of variability in findings across studies within the same outcome domain); (3) indirectness (extent to which the population, intervention, or outcome measure in included studies corresponded to the review question); and (4) imprecision (small sample sizes, wide confidence intervals where reported, or reliance on a single study). Each criterion was evaluated outcome-domain by outcome-domain; where two or more criteria applied, certainty was rated as Very Low. Where only one criterion applied, certainty was rated as Low. The reasoning for each rating is provided within the relevant outcome subsection in the Findings.

**Table 3:** Narrative synthesis summary.

Outcome	k	*n*	Design(s)	Activity type(s)	Direction of findings	Confidence in evidence
Physical wellbeing	1	191 (quant)	Non-rand. Control group	Sports (basketball, archery, table tennis, dart)	Positive (↑ physical, psychological, social WB; *p* < 0.001)	Very low—single study, no RCT, no validated physical measure
Social skills/behaviour	1	92 (quant)	Non-rand. Control group	Group fitness/games	Mixed: negative behaviour ↓ (*p* < 0.001); prosocial NS (*p* = 0.96)	Very low—single study; differential group effect unexplained
Social wellbeing	1	191 (quant)	Non-rand. Control group	Sports	Positive (*p* < 0.001)	Very low—same study as physical WB
School adaptation	5	N = 10 + 20 + 83 + 20 + 10 qualit. & quant.	Qualitative (×4) + Descriptive survey	Sports, cultural, STEM, ECA	Mixed: practitioner-reported positive; survey reports low participation	Very low—mainly qualitative; no shared outcome instrument
Social integration in sports	1	32 (quant)	Non-rand. One-group	Badminton	Positive overall (*p* = 0.04); Turkish > Syrian post-hoc (*p* = 0.03)	Very low—single small-*n* study; asymmetric benefit
Social adaptation	3	20 + 109 + 50 (quant)	Non-rand. One-group (×2) + post-test only	STEM, multi-sport, dodgeball	Inconsistent: one ↑ (*p* = 0.03); one NS (*p* = 0.63); Turkish > Syrian (*p* = 0.028)	Very low—heterogeneous instruments, no pooling possible
School attachment	2	32 + 136 (quant)	Non-rand. One-group + control group	STEM, team sports	Mostly positive (*p* = 0.001–0.005); some subscales NS	Low—2 studies, different activity types; subscale pattern noteworthy
Psychological wellbeing	2	191 quant + 6 qualit.	Non-rand. Control group + focus group	Sports/various ECA	Positive (quant *p* < 0.001; qual: protective factor cited)	Very low—single quant study + counselor perspective only
Attitudes toward school	1	32 (quant)	Non-rand. One-group	STEM	Overall ↑ (*p* = 0.009); risk-preventor subscale strongest (*p* < 0.001); others NS	Very low—single study
Life satisfaction	1	136 (quant)	Non-rand. Control group	Team sports	Positive for Syrian exp. group (*p* < 0.001); Turkish NS	Very low—single study
Self-esteem	1	50 (quant)	Non-rand. One-group	Dodgeball tournament	NS across all subscales (*p* = 0.15)	Very low—single study, null result

### Findings

This section presents the findings of the narrative synthesis across 15 included studies. The results are organized into three subsections: (1) a summary table of outcomes by domain, (2) a detailed outcome-by-outcome synthesis covering physical and psycho-social effects, and (3) a cross-cutting analysis of sources of heterogeneity and the integration of qualitative and quantitative evidence. The synthesis covers 11 distinct outcome domains, each examined through the lens of intervention type, participant characteristics, and methodological quality as assessed by the MMAT and GRADE-adapted certainty ratings. One cross-cutting pattern warrants particular attention before the outcome-by-outcome analysis: across several studies that included both refugee and host-country students, refugee students tended to show greater absolute improvements than their Turkish peers, most notably in life satisfaction (Sağın, 2022), school attachment, and social wellbeing. This differential is most plausibly attributable to lower baseline levels among refugee students rather than to differential responsiveness to the interventions themselves (Yılmaz, 2021). While encouraging in isolation, this pattern also raises a distributional question: whether interventions are closing or merely bridging pre-existing gaps in wellbeing between the two groups. This question is examined systematically in the relevant outcome subsections below and in the Discussion.

### Narrative synthesis summary by outcome

The analysis covers diverse outcomes ranging from physical wellbeing to social integration and school attachment. A total of 11 distinct outcome domains were identified from the reviewed literature. Table 3 presents a synthesized overview of the included studies, categorized by outcome domains, participant characteristics, and the direction of findings.

As shown in the table, physical and psychological wellbeing outcomes were predominantly positive, whereas social behaviours and school adaptation yielded mixed or inconsistent results. The table highlights a significant reliance on quantitative data from non-randomized designs, with participant numbers varying significantly across studies (ranging from *n* = 32 to *n* = 191). The total sample size across qualitative studies was 105, comprising teachers, school principals, and psychological counselors; no included qualitative study directly involved refugee students as primary informants, with the exception of Güvenç (2020). Quantitative studies totaled 1,677 participants and included Turkish and Syrian refugee students across primary, middle, high school, and undergraduate levels. The age range of participants across quantitative studies spanned 7–22 years, encompassing primary school children through university students. This developmental breadth represents a significant constraint on synthesis: the mechanisms through which ECAs promote school attachment and wellbeing differ substantially across developmental stages. For younger children, adult mentorship and structured safety are primary mechanisms; for adolescents, peer belonging and identity development are paramount; for young adults, community integration takes precedence. Thirteen studies included gender information; males were generally overrepresented relative to females. Syrian participants dominated samples; refugees from other national backgrounds (Afghanistan, Iraq) were rarely disaggregated. Three studies did not report participant ethnicity (Özbek, 2023; Tosun et al., 2018; Yaşar et al., 2018). No included study reported data on participants’ length of residency in Turkey, Turkish language proficiency, or socioeconomic status—variables that are plausible moderators of extracurricular benefit and whose omission constitutes a significant gap in the evidence base. The “Confidence in Evidence” column underscores a critical need for more rigorous, large-scale studies in this field, as most current evidence is rated as very low. Notably, across several studies, refugee students appeared to derive greater benefits from extracurricular participation compared to host-country students, suggesting a potential differential impact based on baseline characteristics.

Overall, the methodological quality of included studies substantially limits the interpretability of findings. As indicated by the MMAT appraisal (Table 2), all quantitative studies were non-randomized and the majority were classified as high risk of bias, primarily due to lack of group equivalence (N1) and absence of confounder control (N4). Consequently, the observed effects cannot be interpreted as causal and may reflect pre-existing group differences. It is important to be explicit about what this means for the claims made in this review: the theoretical framework applied here—the integration–inclusion distinction—serves as an interpretive lens for organising and contextualising the available evidence, not as a mechanism for elevating its evidentiary weight. The strength of theoretical interpretation in the Discussion is calibrated to the strength of the underlying evidence: where only a single high-risk study is available, findings are described as preliminary or directional; where a pattern is observed across multiple studies with converging qualitative evidence, somewhat stronger interpretive language is used, while still acknowledging the ceiling imposed by non-randomised designs. Readers should understand this review’s theoretical contribution as offering a productive analytical framework for future research rather than as drawing settled empirical conclusions from the current evidence base. Although effect sizes (e.g., Cohen’s d, η2) were planned to be calculated, this was not possible for the majority of studies due to insufficient statistical reporting (e.g., lack of means, standard deviations, or test statistics). Consequently, the synthesis primarily relies on reported *p*-values and direction of findings, which limits the interpretation of practical significance.

### The effects of Extracurriculars on refugee students

#### Physical effects

Physical effects of extracurricular participation were examined in only one study. Kök (2019) employed a quasi-experimental control-group pretest-posttest design with 191 Syrian high school refugees (experimental *n* = 98, control *n* = 93) who participated in eight weekly four-hour sessions of basketball, archery, table tennis, and dart activities. Refugee students in the experimental group showed statistically significant improvements in physical wellbeing (*p* < 0.001), psychological wellbeing (*p* < 0.001), and social wellbeing (*p* < 0.001), as measured by the Leisure Benefit Scale (Ho, 2008). Significant differences between experimental and control groups were also found on all three dimensions. It should be noted that the Leisure Benefit Scale measures perceived rather than objective physical wellbeing (e.g., self-reported feelings of physical health), and no physiological measures (e.g., fitness tests, body composition) were included. The statistical significance of all outcomes at *p* < 0.001 is atypical for behavioural research and may reflect ceiling or floor effects in the instrument or issues in statistical reporting. These findings should therefore be interpreted with considerable caution. Certainty of evidence was rated as very low due to the high risk of bias, evidence derived from a single study, and use of self-reported rather than objective physical measures, limiting validity. This finding is based on a single study and should be interpreted as preliminary.

#### Psycho-social effects

##### Social skills

One study (Yaşar et al., 2018; *n* = 92) reported significant reductions in negative social behaviours of refugee students (*p* < 0.001) following a 10-week group fitness program, but no significant improvements in prosocial behaviours (*p* = 0.96 > 0.05). Turkish experimental students showed the reverse pattern. The non-significant prosocial finding should be interpreted cautiously, given the small sample size and reliance on a single study. Although the *p*-value was close to 1.00, this finding does not by itself establish the absence of a prosocial effect, as smaller effects may not have been detectable with the available sample. Given the small sample sizes, the studies may have been underpowered to detect moderate effects. The asymmetric pattern—whereby refugee students improved on negative behaviour reduction but not prosocial promotion, while Turkish students showed the reverse—warrants further investigation with designs that can test group-by-outcome interaction effects. Certainty was rated as very low due to high risk of bias, reliance on a single small study, and inconsistent effects across sub-dimensions (negative vs. prosocial behaviours). From an educational psychology perspective, prosocial behaviour is a fundamental socio-emotional catalyst for peer integration and institutional belonging. The non-significant improvement in this domain among refugee students may indicate that prosocial development requires culturally responsive facilitation and specialized instructional scaffolding rather than generic physical activities. This underscores the necessity of aligning extracurricular designs with the specific psycho-social needs and cultural schemas of displaced learners to foster meaningful social participation.

##### Social wellbeing

Kök (2019) reported significant improvement in social wellbeing as part of the Leisure Benefit Scale (*p* < 0.001). As noted in the Physical Effects section above, the study’s uniformly zero *p*-values are atypical and warrant caution regarding the reliability of statistical reporting. Nevertheless, the theoretical mechanism—that cooperative sport creates contexts for skills utilization and peer interaction—is plausible and consistent with international evidence (Ehnold et al., 2024). Certainty was rated as very low due to evidence derived from a single study with atypical *p*-values, no validated social wellbeing measure independent of perceived leisure benefit, and absence of effect sizes.

##### School adaptation

Multiple studies addressed school adaptation with heterogeneous findings. Tosun et al. (2018) reported that most refugee students had difficulty adapting to school and demonstrated low extracurricular participation rates. Teachers in Abay and Güllüpinar (2020) and Arslan and Görgülü (2025), and also school principals in Koşak and Atasoy (2022) advocated extracurricular activities as adaptation strategies. These qualitative practitioner accounts constitute evidence of perceived utility rather than demonstrated effectiveness. No included study used a validated school adaptation instrument in a controlled design. Certainty was rated as very low due to the absence of controlled quantitative evidence, reliance on qualitative perceptions, and lack of standardized outcome measures, limiting comparability across studies.

##### Social integration in sports

The largest study in this review, Özbek (2023; *n* = 1000), found no significant association between folk dance ECA participation and social integration in sports. This null finding from a sample dwarfing all other studies in the review should carry substantial interpretive weight when evaluating positive findings from smaller studies. Alkhlıf (2022) reported significant overall improvement in social integration in sports following 8 weeks of extracurricular badminton participation (*p* = 0.04) among 32 students. However, post-intervention scores remained significantly higher among Turkish students than Syrian students (*p* = 0.03). Given the very small sample size (*n* = 16 per group), these findings should be interpreted cautiously, as small studies are vulnerable to imprecision and unstable effect estimates. The observed group differences therefore cannot be interpreted as evidence that extracurricular activities consistently promote equitable social integration outcomes across participant groups.

Certainty of evidence was rated as very low due to high risk of bias, reliance on a single small study, and limited precision. The asymmetric outcome pattern—where host-country students demonstrated comparatively stronger post-intervention integration scores—suggests the possibility that generic extracurricular programmes may not benefit refugee and host-country students equally under all implementation conditions. From an educational equity and school climate perspective, this finding is noteworthy because social integration is closely associated with psycho-social wellbeing and peer connectedness in school settings. These results may therefore indicate the importance of culturally responsive facilitation and inclusion-oriented programme design when implementing extracurricular activities in refugee-inclusive educational contexts.

##### Social adaptation

Findings on social adaptation were inconsistent across three studies. Arısoy et al. (2022) reported a significant increase following STEM activities (*p* = 0.03; *n* = 20). Yılmaz (2023) reported non-significant findings following a dodgeball tournament (*p* = 0.63; *n* = 50). Yılgın et al. (2022) found Turkish students improved significantly more than Syrian students following 16 weeks of multi-sport activities (*p* = 0.028; *n* = 109). This asymmetric outcome mirrors the pattern observed in social integration findings (Alkhlıf, 2022) and shows that host-country students who already possess established peer networks and language competency may leverage structured sport participation more efficiently for social adaptation gains, while refugee students face additional barriers that attenuate the benefit. These inconsistent findings across studies using the same instrument (Social Adaptation Scale) suggest that outcome variability may be attributable to moderating factors—activity type, duration, and sample composition—rather than a true effect or null. Certainty was rated as very low due to high risk of bias across all included studies, heterogeneity in interventions and measurement tools, and inconsistent findings, precluding robust conclusions.

##### School attachment

Two studies examined school attachment using validated instruments. Sağın (2022; *n* = 136) reported significant improvements in overall school attachment (*p* = 0.001), attachment to teachers (*p* = 0.022), and attachment to classmates (*p* = 0.002) following 10 weeks of mixed-nationality team sports. Güvenç (2020; *n* = 32) reported significant improvement in attachment to teachers (*p* = 0.005) following STEM activities, but non-significant findings for schoolmates (*p* = 0.15). The convergence across two studies with different activity types is noteworthy. One caveat: Sağın (2022) contributes data to both the school attachment domain and the life satisfaction domain (see below), so these two domains are not fully independent. The subscale differences—team sports strengthening peer bonds, STEM strengthening teacher bonds—is theoretically interpretable: team sports necessitate peer interdependence, while STEM activities involve closer adult mentorship. However, both studies are non-randomized with small samples. Certainty was rated as low due to consistent positive findings across two studies, but downgraded due to non-randomized designs, small sample sizes, and lack of confounder control. The School Attachment Scale used by Güvenç (2020) was originally developed by Hill (2006) and adapted for Turkish contexts; its independent use across two studies with convergent results strengthens construct validity for this outcome domain.

##### Psychological wellbeing

Kök (2019) reported significant psychological wellbeing improvement (*p* < 0.001). Bozdağ (2020) identified extracurricular participation as a community-based protective factor from psychological counselors’ perspectives. However, it is based on counselor perception rather than direct child assessment, and no validated mental health instrument was used in either study. Certainty was rated as very low due to reliance on a single quantitative study with high risk of bias, supported only by indirect qualitative evidence from counselors, and absence of validated mental health measures.

##### Attitudes toward school

Güvenç (2020) reported significant overall improvement in attitudes toward school (*p* = 0.009), with a particularly strong effect on perception of school as a risk-preventor (*p* < 0.001). Non-significant improvements were found for school as personal development supporter (*p* = 0.14) and school as appealing setting (*p* = 0.18). The pattern—where school is perceived primarily as a place of safety rather than development or appeal—aligns with educational psychology frameworks on secure learning environments. These frameworks identify psychological safety as a structural precondition for cognitive engagement and peer belonging rather than an end in itself. STEM activities may address this by creating structured, predictable, engaging environments that begin to bridge from safety toward development. Certainty was rated as very low due to evidence derived from a single non-randomized study (high risk of bias), a small sample (*n* = 32), absence of a control group for the attitudinal outcome, and lack of effect sizes. This finding is based on a single study and should be interpreted as preliminary.

##### Life satisfaction

Sağın (2022) reported a significant increase in life satisfaction for Syrian refugee students in the experimental group (*p* < 0.001) with non-significant findings for Turkish students. The observed differences in outcomes align with a condition well-documented among Syrian refugees in Turkey (Yılmaz, 2021)—whereby those with lower baseline wellbeing possess a greater margin for positive transformation through extracurricular interventions. Nevertheless, this remains a single study. Certainty was rated as very low due to evidence derived from a single non-randomized study, potential baseline differences between groups, and lack of reported effect sizes. This finding is based on a single study and should be interpreted as preliminary.

##### Self-esteem

Yılmaz (2023) reported non-significant findings in overall self-esteem (*p* = 0.15) and across all subscales following an eight-session dodgeball tournament (*n* = 50). Given the relatively small sample size, the study may have been insufficiently powered to detect small-to-moderate effects, and the null findings should therefore be interpreted cautiously rather than as conclusive evidence of no impact. Certainty of evidence was rated as very low due to high risk of bias, imprecision related to the limited sample size, and reliance on a single study. These findings should accordingly be regarded as preliminary.

Taken together, the outcome-level findings reveal a pattern of selective and context-dependent benefits rather than uniformly positive effects. Positive findings were most consistent in domains related to school attachment, psychological wellbeing, and perceived social wellbeing; however, these results derive predominantly from non-randomized studies with high risk of bias and therefore cannot be interpreted causally. Outcomes related to social adaptation, social skills, and school adaptation showed substantial variability across studies. A recurrent pattern across all outcome domains was that certainty of evidence remained low to very low, with no domain supported by high-certainty evidence.

Variation in findings across studies appears to be associated with several potential moderating factors. First, intervention duration may play an important role, as longer-term programs (e.g., 10–16 weeks) appeared to demonstrate more consistent positive outcomes compared to short-term or one-off interventions. Second, the type of extracurricular activity may influence the nature of outcomes: sport-based activities appeared more likely to facilitate peer interaction and social integration, whereas STEM-based activities appeared more closely associated with school attachment, particularly in relation to teachers. Third, participant background (refugee vs. host-country students) emerged as another possible moderator, as refugee students in some studies demonstrated greater relative improvements, potentially reflecting lower baseline levels or greater scope for change. These interpretations should be understood as exploratory rather than confirmatory, as none of the included studies formally tested moderation effects.

The integration of qualitative and quantitative findings reveals a complementary pattern. Quantitative studies generally report positive or mixed effects of extracurricular activities on outcomes such as school attachment, psychological wellbeing, and social integration. These findings are supported by qualitative evidence, in which teachers, school principals, and psychological counselors consistently describe extracurricular activities as important tools for facilitating adaptation, social interaction, and emotional support among refugee students. Qualitative data further enrich the interpretation of quantitative results by highlighting the mechanisms through which these effects may occur, particularly emphasizing the role of structured social interaction, safe environments, and opportunities for participation. However, the integration remains limited by the absence of direct student perspectives in most qualitative studies and the lack of studies explicitly designed to combine qualitative and quantitative approaches. As a result, the observed alignment should be interpreted as suggestive rather than confirmatory. Importantly, findings should be interpreted as context-sensitive rather than representing a unified effect, given the substantial heterogeneity in participant age ranges (7–22 years), intervention types, outcome measures, and study designs across the 15 included studies.

## Discussion

The theoretical interpretation offered in this review should be understood as heuristic rather than confirmatory, given the low certainty and heterogeneity of the underlying evidence base. The integration–inclusion distinction serves as a useful interpretive lens rather than a definitive explanatory model, and the interpretations that follow are strictly calibrated to the strength of the available evidence. Vislie (2003) argues that integration assumes a deficit in the student and tasks the individual with fitting into existing structures, while inclusion demands a systemic restructuring of the educational environment to genuinely accommodate all learners. Hausstätter and Jahnukainen (2014) extend this analysis by showing that integration-oriented reforms tend to focus on placing marginalised students within mainstream settings without fundamentally reforming the pedagogical conditions they encounter there. Read through this lens, and acknowledging the low certainty of the synthesized literature, the evidence reveals a pattern tentatively indicative of integration-oriented rather than inclusion-oriented conditions, as associations cluster around individual-level gains and physical co-presence while systemic equity-transformative features remain largely unexamined. The socio-emotional and physical outcomes within the Turkish context may tentatively illustrate this integration logic most clearly. For instance, Kök (2019) reported significant improvements in perceived physical wellbeing following structured sport participation, a finding that suggests sport may be functioning primarily as a stabilising tool for individual students. This aligns with the compensatory logic that Vislie (2003) identifies as characteristic of integration. Similarly, qualitative evidence from Bozdağ (2020) frames extracurricular participation as a protective factor for psychological resilience, which is consistent with international evidence regarding resilience-building mechanisms (Abdi et al., 2023; Godor et al., 2024). However, as resilience-oriented interventions are often individually targeted, they may help the student cope with an existing environment rather than transforming the structural conditions of that environment. As Dryden-Peterson (2024) argues, persistent dilemmas remain regarding alienation from curriculum and structural misalignment in refugee education. To the extent that these conditions hold, ECAs designed primarily as resilience resources may address individual-level outcomes without necessarily engaging the institutional conditions that constrain equitable participation.

Moving from individual to relational outcomes, the school attachment evidence represents the closest that the included studies come to an inclusive mechanism. Two studies converged in reporting significant improvements in attachment: Sağın (2022) found that mixed-nationality team sports strengthened peer attachment, while Güvenç (2020) found that STEM activities strengthened student–teacher attachment. Research confirms that these structured interactions are the primary domains through which attachment is built (Abdi et al., 2023; Molla, 2024). Furthermore, Anderson et al. (2024) describe teachers as “boundary workers” who broker a sense of welcome for refugee-background young people. When STEM activities build these bonds, they may approach an inclusive function by creating relational conditions where the student experiences the school as genuinely responsive. However, the strongest effect in Güvenç's (2020) study emerged for the perception of school as a risk-preventor, while subscales for personal development and school appeal were non-significant. This pattern suggests that the activities may not yet have produced the fuller experience of school as a belonging-oriented space that characterises inclusion in Vislie’s (2003) framing.

Although constrained by the low overall certainty of the underlying evidence, some of the most theoretically notable preliminary patterns emerge in the reported social integration and adaptation dynamics. In primary studies such as Yılgın et al. (2022), host-country Turkish students improved significantly more than Syrian refugee students (Alkhlıf, 2022; Yılgın et al., 2022). Additionally, Yaşar et al. (2018) found that refugee students showed reductions in negative social behaviour but no improvement in prosocial behaviour, while Turkish students showed the reverse. This pattern might be tentatively interpreted throughsocial capital theory, where Ehnold et al. (2024) distinguish between bonding and bridging social capital, noting that such activities frequently strengthen bridging capital for already-advantaged host-community members while producing modest gains for newcomers. ECAs that are mixed in participation but unmodified in design might carry the risk ofreinforcing host culture norms, potentially placing students with lower social and linguistic capital at a disadvantage. An equity-oriented design would ideally attend to the structural sources of disadvantage—such as social isolation and linguistic barriers—rather than relying solely on the magnitude of disadvantage to generate statistically detectable improvements.

Age-related developmental differences should also be considered when interpreting the synthesized findings. The included studies covered a broad age range (7–22 years), spanning childhood, adolescence, and young adulthood. Because developmental needs, peer relationships, social–emotional regulation, and responses to extracurricular interventions may differ substantially across these stages, synthesizing outcomes across the full age range may obscure age-specific mechanisms. Accordingly, the findings should not be interpreted as indicating uniform intervention effects across developmental stages. Future research should examine outcomes within more developmentally homogeneous age groups and explicitly test whether intervention effects vary by developmental stage.

The current evidence relies heavily on practitioner-derived data (e.g., reports from teachers, principals, and counselors), which reflects institutional appraisals of inclusion rather than the lived experiences or perceived barriers of the students themselves—a critical divergence highlighted in recent methodology literature (Anderson et al., 2024). Consequently, without student-reported socio-emotional or academic adjustment measures, the most pedagogically significant outcomes remain unmeasured.

Furthermore, while the integration-versus-inclusion and social capital frameworks offer valuable analytical heuristics, the resulting systemic inferences must be interpreted with caution. Given the low to very low overall certainty of evidence, the identified structural barriers represent directional trends rather than definitive diagnostics. Future research should therefore prioritize participatory, student-centered approaches to capture refugee youths’ lived experiences. Although the proposed recommendations for co-construction and equity audits remain tentative, they offer valuable working hypotheses to bridge the policy-practice gap in refugee education.

## Limitations

A major limitation of the current synthesis is the absence of effect size reporting in primary studies. This prevented quantitative comparison of intervention impact and restricted interpretation to statistical significance rather than magnitude of effect.

Second, although grey literature was actively sought, the possibility of publication bias cannot be ruled out. Studies with null or negative findings are less likely to be published or indexed in the databases searched, which may inflate the apparent positive effect of extracurricular activities in this review.

A potential limitation concerns the structural asymmetry in search string design between languages. While the English strategy relied on broad umbrella terminology (*“extracurricular”*), the Turkish search strings incorporated activity-specific terms (e.g., sports, arts, school clubs) to accommodate national indexing patterns. Although this adaptation aimed to maximize sensitivity within national databases, it is possible that English-language studies focusing strictly on specific activities without utilizing generic extracurricular descriptors were not captured.

The majority of qualitative evidence was drawn from the perspectives of teachers, school principals, and psychological counselors rather than from refugee students themselves. This practitioner-centred viewpoint may not capture the lived experiences, preferences, or barriers perceived by students, and constitutes a critical limitation for educational research, where student voice and agency are central to understanding the subjective learning experience. The complete absence of validated, student-reported socio-emotional or academic adjustment instruments across nearly all included studies means that the most pedagogically significant outcomes—from the perspective of the child—remain unmeasured in this literature. This gap obscures the student-centered reality of school integration and limits the ability to assess the true impact of educational interventions on learner engagement. Future research must prioritise direct data collection from refugee students using validated wellbeing instruments. The reliance on self-reported measures for physical wellbeing limits the extent to which these findings can be interpreted as evidence of objective physical change. Future studies should incorporate objective indicators of physical health and fitness alongside self-reported measures.

The framework coding relied on reported study characteristics; therefore, the absence of reported adaptations or facilitator training should not necessarily be interpreted as the absence of inclusive practices in implementation. None of the included studies reported data on key moderating variables—including Turkish language proficiency, length of residency, and socioeconomic status—that plausibly affect the magnitude of extracurricular benefit. The absence of these covariates prevents adjustment for confounding and limits causal interpretation across all outcome domains. Nine of the 15 included studies are unpublished theses or dissertations. While assessed with the same MMAT criteria as peer-reviewed publications, the absence of independent editorial and blind peer review scrutiny means that reporting completeness in these studies may reflect institutional thesis requirements rather than independent methodological verification.

## Conclusion

This systematic review examined the effects of extracurricular activities on various developmental outcomes for refugee students in Turkey, evaluating these links through the integration–inclusion distinction. The synthesis of 15 studies published between 2015 and 2026 indicates that while ECA participation is tentatively associated with positive trends—most consistently in school attachment and socio-emotional adjustment—the certainty of evidence remains low to very low across all domains. A substantive finding of this review is the asymmetric distribution of these associations: in mixed-group designs, host-country students appear to demonstrate stronger gains in social integration than refugee students. This pattern, rendered legible through the integration–inclusion framework, suggests that the observed associations may reflect an integration-oriented logic—characterised by physical co-presence and individual stabilisation—rather than a fully inclusion-oriented framework that would require a structural reconfiguration of institutional norms.

While the empirical scope of the included studies remains focused on local implementation, these findings highlight potential tensions in practice, suggesting that without an explicit equity focus, the links between participation and social outcomes may inadvertently align with assimilationist patterns unless activities are intentionally designed to be inclusive. ECAs do not represent a causal solution to the complex challenges of displacement; however, they constitute a vital institutional space where educational institutions can move beyond mere accommodation toward genuine educational equity.

To advance the understanding of these complex associations, future research should transition from simple outcome measurement to equity-centered methodologies. We propose: Implementing randomised or quasi-experimental designs to move beyond reported associations and identify for whom these activities are most effective; Prioritising validated, student-reported instruments to capture the child’s subjective experience; Distinguishing between primary, secondary, and higher education populations to identify age-specific associative patterns; Positioning refugee students as active co-constructors of ECA content to ensure that participation is associated with genuine belonging rather than passive attendance.

Ultimately, as the current evidence base overwhelmingly reflects Syrian populations, these tentative associations should be generalised with caution. This review concludes that while these 15 studies do not evaluate national policy frameworks directly, their collective findings suggest that achieving inclusion in refugee education requires more than shared physical space; it demands a broader commitment to transforming the immediate educational environments in which refugee youth participate.

## Data Availability

All data supporting the findings of this systematic review are available within the manuscript and its supplementary materials.
